# Feasibility of selective cardiac ventricular electroporation

**DOI:** 10.1371/journal.pone.0229214

**Published:** 2020-02-21

**Authors:** Alan Sugrue, Vaibhav R. Vaidya, Christopher Livia, Deepak Padmanabhan, Anas Abudan, Ameesh Isath, Tyra Witt, Christopher V. DeSimone, Paul Stalboerger, Suraj Kapa, Samuel J. Asirvatham, Christopher J. McLeod

**Affiliations:** 1 Division of Heart Rhythm Services, Department of Cardiovascular Diseases, Mayo Clinic, Rochester, MN, United States of America; 2 Department of Cardiovascular Medicine and Department of Molecular Pharmacology and Experimental Therapeutics, Center for Regenerative Medicine, Mayo Clinic, Rochester, MN, United States of America; 3 Division of Pediatric Cardiology, Department of Pediatric and Adolescent Medicine, Mayo Clinic, Rochester, MN, United States of America; University of Minnesota, UNITED STATES

## Abstract

**Introduction:**

The application of brief high voltage electrical pulses to tissue can lead to an irreversible or reversible electroporation effect in a cell-specific manner. In the management of ventricular arrhythmias, the ability to target different tissue types, specifically cardiac conduction tissue (His-Purkinje System) vs. cardiac myocardium would be advantageous. We hypothesize that pulsed electric fields (PEFs) can be applied safely to the beating heart through a catheter-based approach, and we tested whether the superficial Purkinje cells can be targeted with PEFs without injury to underlying myocardial tissue.

**Methods:**

In an acute (n = 5) and chronic canine model (n = 6), detailed electroanatomical mapping of the left ventricle identified electrical signals from myocardial and overlying Purkinje tissue. Electroporation was effected via percutaneous catheter-based Intracardiac bipolar current delivery in the anesthetized animal. Repeat Intracardiac electrical mapping of the heart was performed at acute and chronic time points; followed by histological analysis to assess effects.

**Results:**

PEF demonstrated an acute dose-dependent functional effect on Purkinje, with titration of pulse duration and/or voltage associated with successful acute Purkinje damage. Electrical conduction in the insulated bundle of His (n = 2) and anterior fascicle bundle (n = 2), was not affected. At 30 days repeat cardiac mapping demonstrated resilient, normal electrical conduction throughout the targeted area with no significant change in myocardial amplitude (pre 5.9 ± 1.8 mV, 30 days 5.4 ± 1.2 mV, p = 0.92). Histopathological analysis confirmed acute Purkinje fiber targeting, with chronic studies showing normal Purkinje fibers, with minimal subendocardial myocardial fibrosis.

**Conclusion:**

PEF provides a novel, safe method for non-thermal acute modulation of the Purkinje fibers without significant injury to the underlying myocardium. Future optimization of this energy delivery is required to optimize conditions so that selective electroporation can be utilized in humans the treatment of cardiac disease.

## Introduction

Electroporation, or electropermeabilization, is a phenomenon in which cell membrane permeability is increased on exposure to pulsed electric fields (PEFs). The delivery of PEFs to tissue can result in both irreversible and reversible effects. While reversible electroporation has been studied since the 1980s[1–4], the concept of irreversible electroporation is relatively new and proposed by Davlaos in 2005[5] in a landmark paper highlighting the power of electric fields to produce tissue ablation without detrimental thermal effects. While much of the initial focus on the irreversible effects of PEFs was oncological, Hong[6] and later Wittkampf[7, 8], explored cardiac applications which lead to the emergence of PEFs as a novel cardiac ablation modality[9–12].

Today, radiofrequency energy is the most common energy modality used for cardiac ablation followed by cryothermal energy. The utilization of PEFs as opposed to current approaches (radiofrequency or cryothermal energy) for targeting arrhythmogenic foci is attractive as current energies for targeting cardiac tissue do not afford multifaceted cell-specific modulation, exhibiting either an all or none phenomenon (radiofrequency), or if they have reversible potential, it only provides mapping opportunities (cryomapping). Specifically, one particular limitation with current cardiac ablation energies is the inability to apply energy on or close to cardiac blood vessels. IRE has shown significant promise in this regard and led largely by the work of Maor[13–16], with all studies showing that the connective matrix of the blood vessels remained intact and the number of vascular smooth muscle in the arterial wall decreased with no evidence of aneurysm, thrombus formation or necrosis. This is a clear improvement over radiofrequency and cryothermal energy.

Recently we have seen the publication of the first in human data on atrial fibrillation PEF ablation with promising results. While there has been considerably preliminary success on atrial tissue, application on cardiac tissue has been limited. Of particular clinical value would be a means to selectively target the cardiac Purkinje system which plays a pivotal role in the genesis and maintenance of multiple different ventricular arrhythmias in both the failing and healthy heart. [17–27] In an acute *ex-vivo* model, PEFs delivered to the ventricular myocardium was shown to be successful in targeting Purkinje tissue[28]. In this study, PEF consistently eradicated all Purkinje potentials at voltages between 750V and 2500V and the ventricular electrogram amplitude was only minimally reduced, but not clinically significant, by ablation; 0.6 mV ± 2.3 mV (p = 0.03). Further PEF resulted in significantly reduced the window of vulnerability to ventricular fibrillation and in 4 hearts following IRE delivery, VF could not be re-induced., we sought, therefore, to further clarify the effect of PEF therapy (irreversible or reversible) on Purkinje fibers in an *in-vivo* model. Through dose-titrated PEF delivery, as well as electroanatomical mapping and histological analysis at both acute and chronic time points, we aimed to examine the effects of high voltage PEFs applied to the myocardium on animal survival, cardiac conduction and ventricular injury.

## Methods

### Animal preparation and experimental setup

The Mayo Clinic Animal Care and Use Committee approved this study involving 11 male mongrel dogs (weighing 30–40 kg). Experiments were carried out under general anesthesia with intubation and mechanical ventilation. We used ketamine (10 mg/kg), and diazepam (0.5 mg/kg) for induction and isoflurane inhalation (1–3%) for maintenance. Core body temperature was maintained at 37°C using a heating pad placed underneath the dog. Vascular access was obtained with the modified Seldinger technique. Standard 9 French sheaths were placed percutaneously in the femoral artery, femoral veins, and the external jugular vein for catheter access to the heart. Heart rate and femoral arterial blood pressure were continuously monitored. Standard electrocardiographic leads and intracardiac electrograms filtered at 30–500 Hz were electronically recorded using a Prucka CardioLab recording system (GE Healthcare, Wauwatosa, Wisconsin). Fluoroscopy and intracardiac ultrasound (Acuson Sequoia; Siemens) were used to navigate the cardiac chambers and to guide catheter manipulation. Detailed three-dimensional electroanatomical mapping of the left ventricle via retro-aortic access was performed using the CARTO^®^ Mapping System (Biosense Webster, Inc., Diamond Bar, CA, USA). Intracardiac mapping was performed using a Navistar^®^ ablation catheter with 3.5 mm tip and electrode spacing of 2–5–2 mm (Biosense Webster). Heparinized saline was used to prevent intraprocedural clotting, aiming for an Activated clotting time (ACT) >300. In the chronic studies, intravenous cefazolin (1000 mg) was given during the procedure followed by oral cefpodoxime (10 mg/kg) post-procedure to prevent infection.

### Electroanatomical mapping

Using the Navistar^®^ ablation catheter, three-dimensional electro-anatomical maps were created, and mapping performed throughout the left ventricle; in addition to the annotation of the His bundle; fascicular and Purkinje signals. Fascicular potentials were labeled if a high frequency electrogram occurred before the ventricular electrogram but with a short isoelectric period intervening. Purkinje potentials (PP), which are primarily from the non-insulated portion of the conduction system and represent electrical activation of Purkinje cells/tissue, were recognized by no isoelectric period between the high-frequency PP and the ventricular electrogram. The left bundle was assumed to be a direct extension of the His potential without any atrial electrogram visible on the proximal pair of electrodes. Repeat mapping was performed at the end study utilizing the orientation of the His and coronary sinus to compare the original mapping and ablation points with the remap of the same region.

### Percutaneous PEF protocol

Before delivery of the PEF, the canines received vecuronium (0.05–0.1 mg/kg) to limit muscle contraction. PEF delivery was synchronized with the QRS and delivered using the NanoKnife^™^ system (AngioDynamics, Queensbury, NY, USA) in a bipolar fashion between modified 8mm radiofrequency ablation catheter at sites identify with our mapping catheter. Based upon on our previous *ex-vivo* success[28], for the chronic studies (n = 6) we delivered ten pulses in a monopolar configuration at voltages between 500-1500V at a frequency of 0.83–1 Hz with a pulse duration of 90 microseconds (μs). For the acute studies (n = 5), except the first canine, we delivered ten pulses at a frequency of 0.83–1 Hz with varying voltages (750-3000V) and a pulse duration of 20 μs. For the first acute canine, we delivered energy at the same frequency and voltage range but with a pulse duration of 90 μs, and delivered between 10–100 pulses. **S1 Table** shows the complete pulsed electric field parameters delivered across all acute and chronic studies. After delivery of the PEF, if a Purkinje signal was still present (at 1 min and 5mins), the voltage was increased and/or pulse duration changed, and repeat PEF delivered to the same location until PP eradication. The 1 min and 5 min time window was selected upon expert opinion. The persistence of PP was defined as persistence at 1 min.

**Fig 1:**
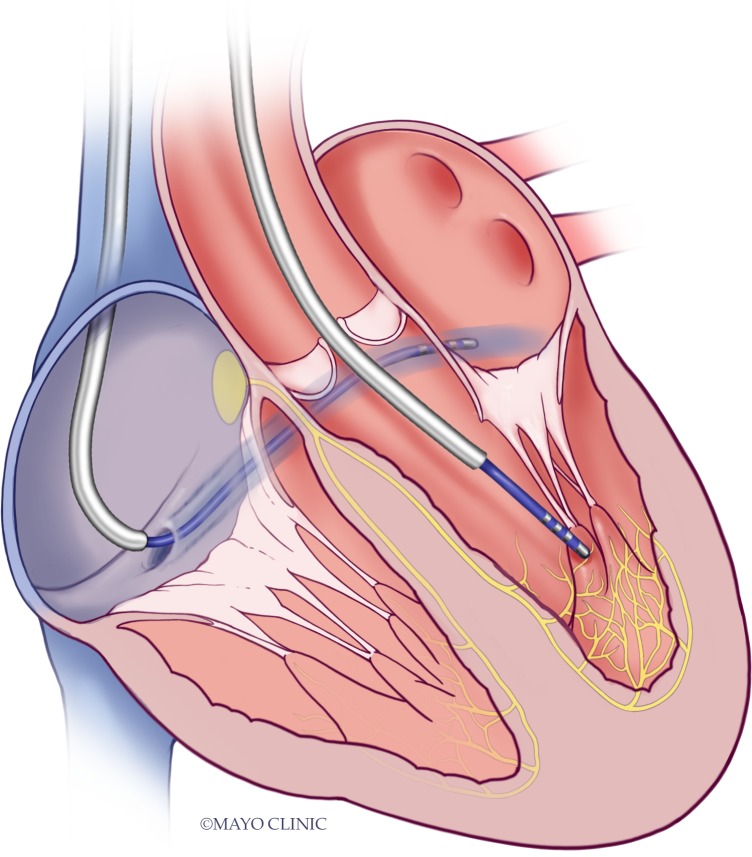
Demonstration of location of the catheters used for pulsed electric field delivery, with one catheter fixed in the coronary sinus and the other located at a Purkinje potential in the LV.

The electric field was delivered between two Boston Scientific 5mm Blazer catheters (EP Technologies, Boston Scientific Corp, Natick, Massachusetts) in a bipolar configuration. The site of delivery was deliberately chosen to be nearby but not directly in contact with the PP mapped using the electroanatomical mapping catheter. The second bipolar catheter was placed in the coronary sinus and remained fixed (**Fig 1**). The distance between the electroporation catheter (Blazer) and the coronary sinus varied depending upon the location of the detected signal; it was, however never more than 3cm away. The mapping catheter was not moved away from the Purkinje during electroporation delivery. After delivery of the PEF the electrogram we observed for loss of the signal and recorded the voltage of the local electrogram immediately after delivery, at one and five minutes.

### Follow‐up study and histologic analysis

The survival period ranged from 19 to 36 days, with a mean of 29 days. The end procedure was performed similarly to the initial procedure although no PEF was delivered. After completing the repeat mapping, animals were sacrificed with electrical induction of ventricular fibrillation. Postmortem gross anatomic examination was performed to evaluate complications and efficacy of the PEF delivered. Histologic samples were obtained after embedding hearts in paraffin for 1–2 weeks after the procedure. After sectioning the heart, histologic staining was performed with hematoxylin and eosin to assess tissue injury and Purkinje fiber damage. Purkinje fibers were identified by visual appearance and location.

### Statistical analysis

Statistical analysis was performed using JMP Pro 13.0.0 software (SAS, Cary, NC). Data are expressed as mean ± standard deviation (SD) unless otherwise stated. Difference between groups was assessed using ANOVA. A p-value <0.05 was considered significant.

## Results

### Pulsed electric field delivery

We delivered a total of 52 PEF therapies across five acute and six chronic canines (range 1–7 per animal) and all animals survived. The electric field magnitude delivered to the endocardium of the heart varied from 750 to 3000V, with 750 V the most commonly delivered (18/52, 35%) followed by 1125 V (10/52, 19%), and 1500 V (8/52, 15%). We mostly delivered a train of ten pulses (47/52, 90%).

### Effects on cardiac conduction tissue

**Fig 2:**
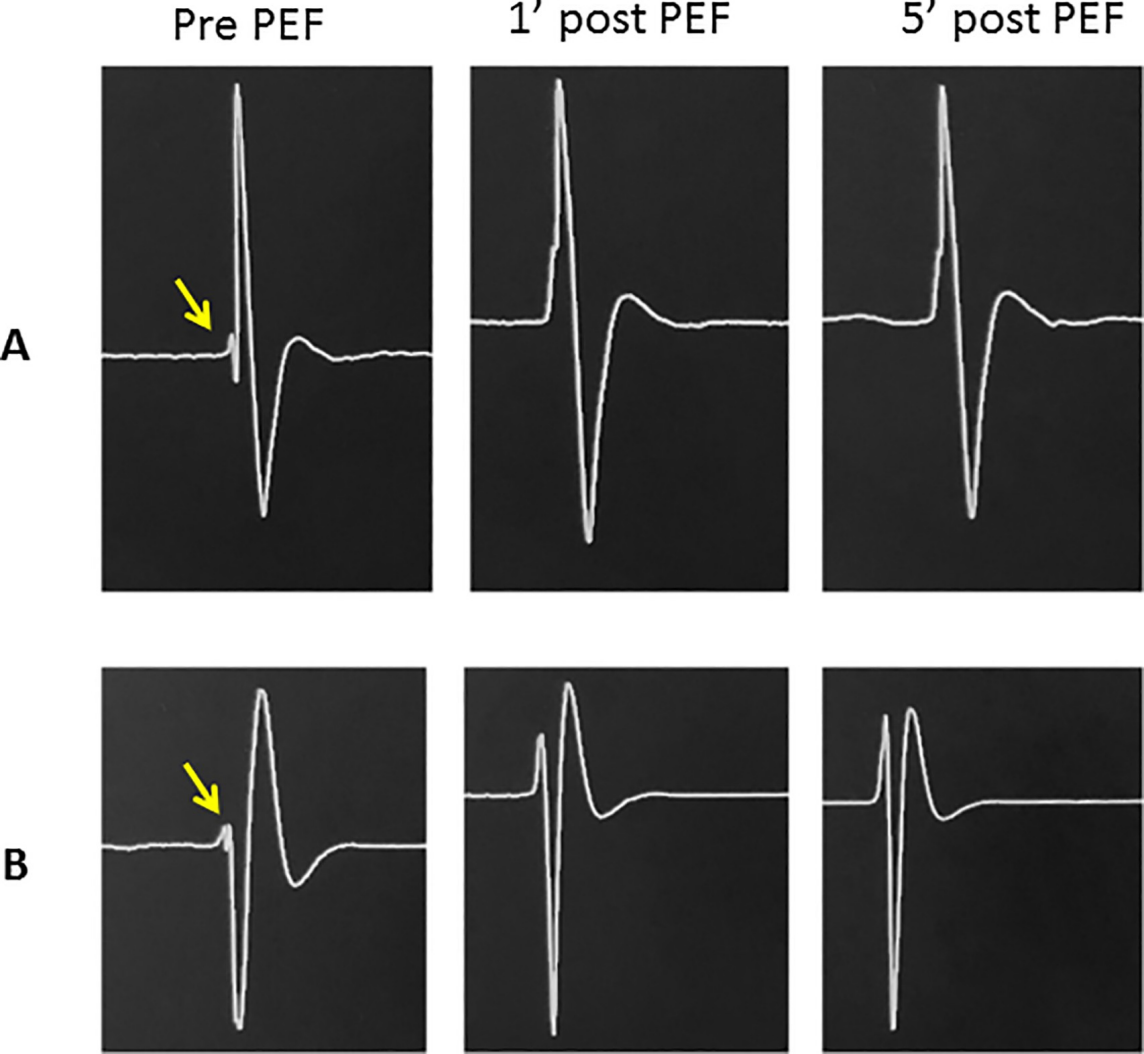
Two examples (A and B) of loss of Purkinje fiber electrical conduction after PEF delivery. Yellow arrow highlights the Purkinje potential.

**Fig 3:**
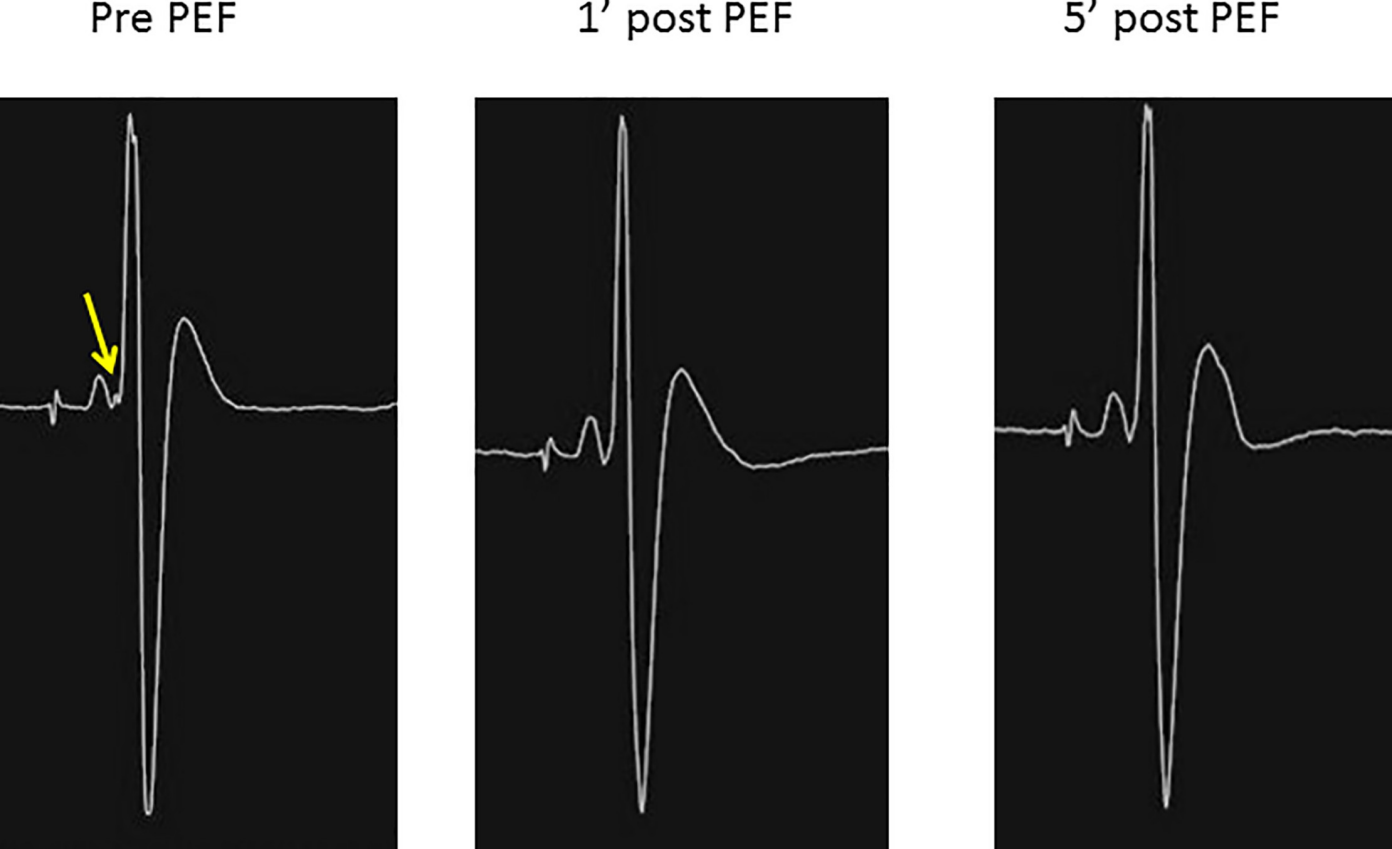
Loss of small Purkinje potential, but resistance of the His to PEF.

**Fig 4:**
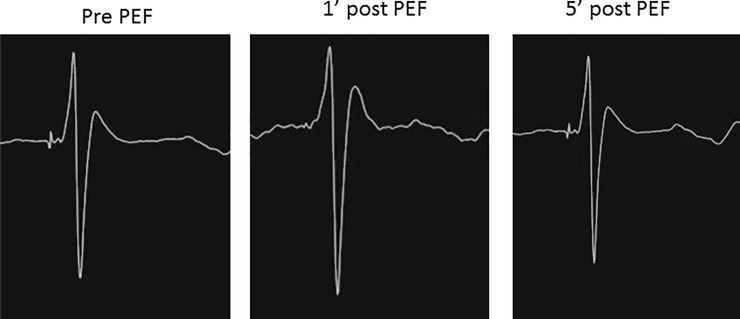
Reversible loss of a fascicular signal from PEF delivery.

In both the acute and chronic animal models, PEF demonstrated an acute dose-dependent effect on the electrical conduction of all left ventricular Purkinje fibers. 4 PEF deliveries did not alter electrical conduction, and in these cases we had attempted PEF delivery to the His (n = 2, acute canine 4, chronic canine 3) and anterior fascicle bundle (n = 2, chronic canine 2). **Fig 2** shows two typical examples of loss of Purkinje signals that we observed after PEF delivery. The His bundle was resistant to the PEF delivered with no change in its signal despite multiple deliveries (**Fig 3**). In the cases of the anterior fascicle, we observed loss of the signal after increasing doses of PEF but then recovery at 5 minutes (**Fig 4**). **Table 1** displays the impact of the PEF delivered on the detected PP at different time points.

**Table 1:** Effect of PEF on Purkinje potential.

Pulse Duration (us)	Voltage (Volts)	Number (n = 52)	Purkinje Potential Present		
			1 min, n (%)	5 min, n (%)	30 days, n (%)
20	750	3	1 (67)	1 (67)	-
	1050	1	1 (100)	1 (100)	-
	1125	2	1 (50)	1 (50)	-
	1500	4	2 (50)	4 (100)	-
	1800	2	0 (0)	0 (0)	-
	2250	6	1 (16)^	1(16)^	-
	3000	2	0 (0)	0 (0)	-
90	500	1	0 (0)	1 (100)	1 (100)
	750	15	5 (33)	6 (40)	9 (100)*
	1050	4	1 (25)^	2 (25)	
	1125	8	0 (0)	1 (13)	8 (100)
	1500	4	2 (50)~	2(25)~	4 (100)

*n = 13 after removing 6 that were acute studies

^signals remaining represents a His bundle signal that could not be targeted

~ signals remaining represents a large anterior fascicle that could not be targeted

### Pulse duration

When the train of pulses was delivered at a duration of 20 microseconds, a threshold of greater than 1800V was required to record persistent acute loss of the PP. In the cases where there was the persistence of the PP after PEF delivery, redelivery at a similar or higher voltage resulted in the loss of the PP. Specific examples from our study were the persistence of the PP at 750V which was successfully targeted with the redelivery of the PEF at 1500V; persistence at 1050V with redelivery at 1500V and then 2250V been successful; 1125V redelivered at 1500V resulting in success and lastly 1500V repeated at 1500V with subsequent success. As we have stated, there was marked resilience of the His bundle to PEF. We attempted PEF delivery to the His at 2250V; however, we were unable to alter the His signal.

When the train of pulses was delivered at 90 microseconds, a voltage greater than 1050V generally resulted in the persistent loss of the PP. Similar to our observation at a 20-microsecond pulse duration, in the cases where there was a persistence of the PP after PEF delivery, redelivery at a similar or higher voltage resulted in the acute loss of the PP. Specifically, at 750V, redelivery of the PEF at 1050V or 1125V, or an increase in the number of pulses delivered from 10 to 20 or 30 resulted in success. At 1050V and 1500V, we were unable to permanently target the His or a large anterior fascicle, respectively.

Using our previously created electroanatomical map, we returned to the exact ablation location in the chronic animals at 30 days and noted that all PP had returned.

### Effect on myocardial amplitude

**Fig 5:**
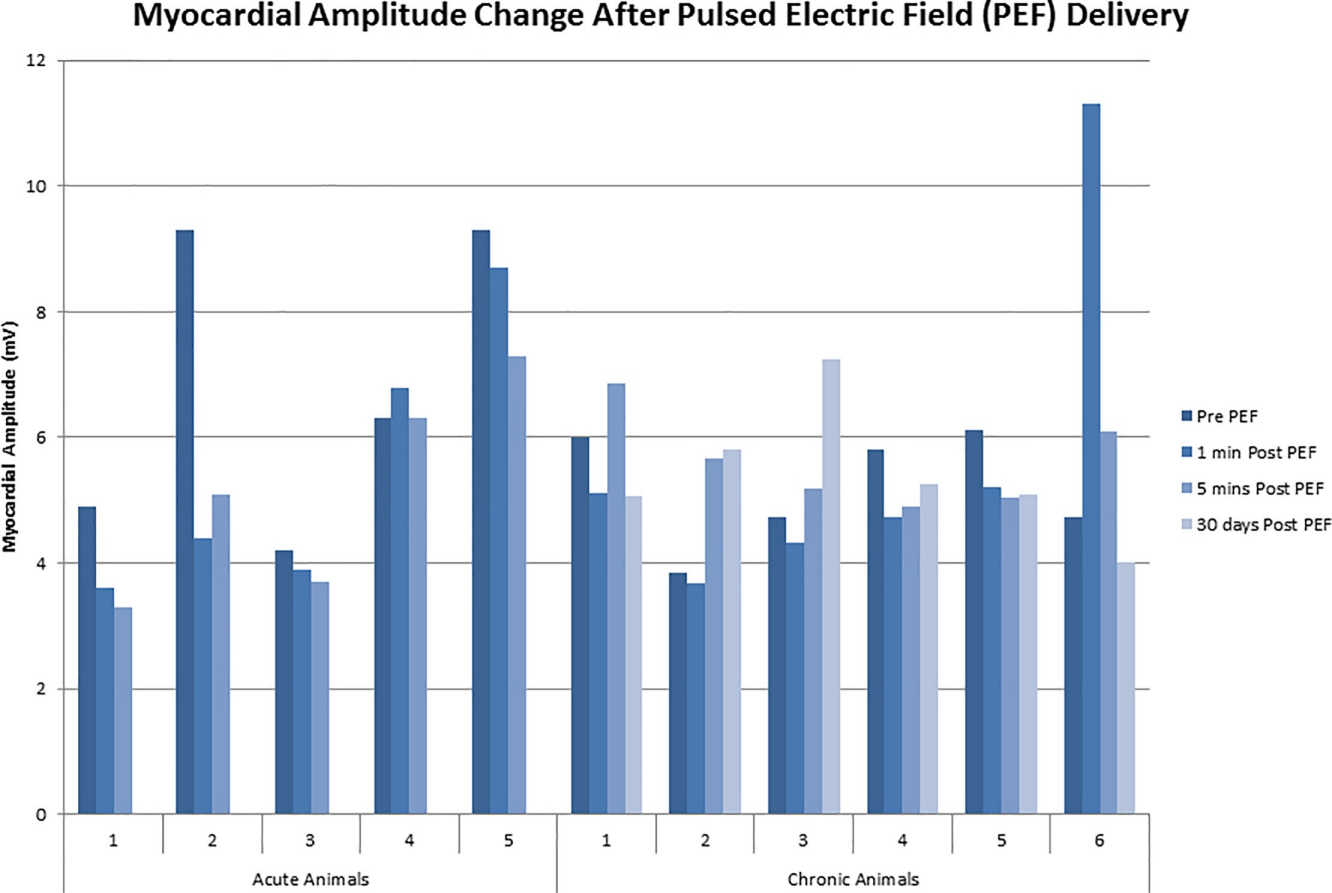
Myocardial amplitude change after Pulsed Electric Field (PEF) delivery.

Across all animal studies, there was no statistically significant change in myocardial amplitude post-PEF delivery, over 1 minute, 5 minutes or 30 days. The mean amplitude pre-PEF delivery was 5.9 ± 1.8 mV, 5.6 ± 32.4 mV 1 min post-PEF delivery, 5.4 ± 1.2 mV 5 min post-PEF and in the chronic animals 5.4 ± 1.0 mV at approximately 30 days (p = 0.921, **Fig 5**). Closer examination of the individual amplitudes at the site of delivery highlights that 1-minute post-PEF, amplitudes rarely changed by ± 1.5mV (n = 9/11, 81%), an effect that was consistent over 5 minutes (n = 8/11, 72%). In one animal (chronic animal 6) the amplitude increased after delivery but then returned to baseline. In another animal (acute animal 2), there was a decrease of 5mV from the baseline amplitude which did not recover over 5mins.

### Effect on cardiac conduction

There were no significant differences detected in QRS duration after acute PEF delivery or at 30 days (**Table 2**). The pre-PEF QRS mean was 72.9± 8.4 msec, 5 mins post-PEF 76.7 ±8.8 msec and at 30 days 75.5 ± 8.1 msec (p = 1.0).

**Table 2:** Change in QRS duration after PEF.

		Pre PEF	5 mins post PEF	30 days of PEF
Acute Animals				
	1	81	85	-
	2	74	78	-
	3	69	65	-
	4	59	65	-
	5	63	69	-
Chronic Animals				
	1	84	86	80
	2	80	80	82
	3	82	84	82
	4	67	74	80
	5	76	89	84
	6	67	69	65
Mean (±SD), ms		72.9± 8.4	76.7 ±8.8	7.8 ± 6.9
ms- milliseconds				

### Histopathological analysis

**Fig 6:**
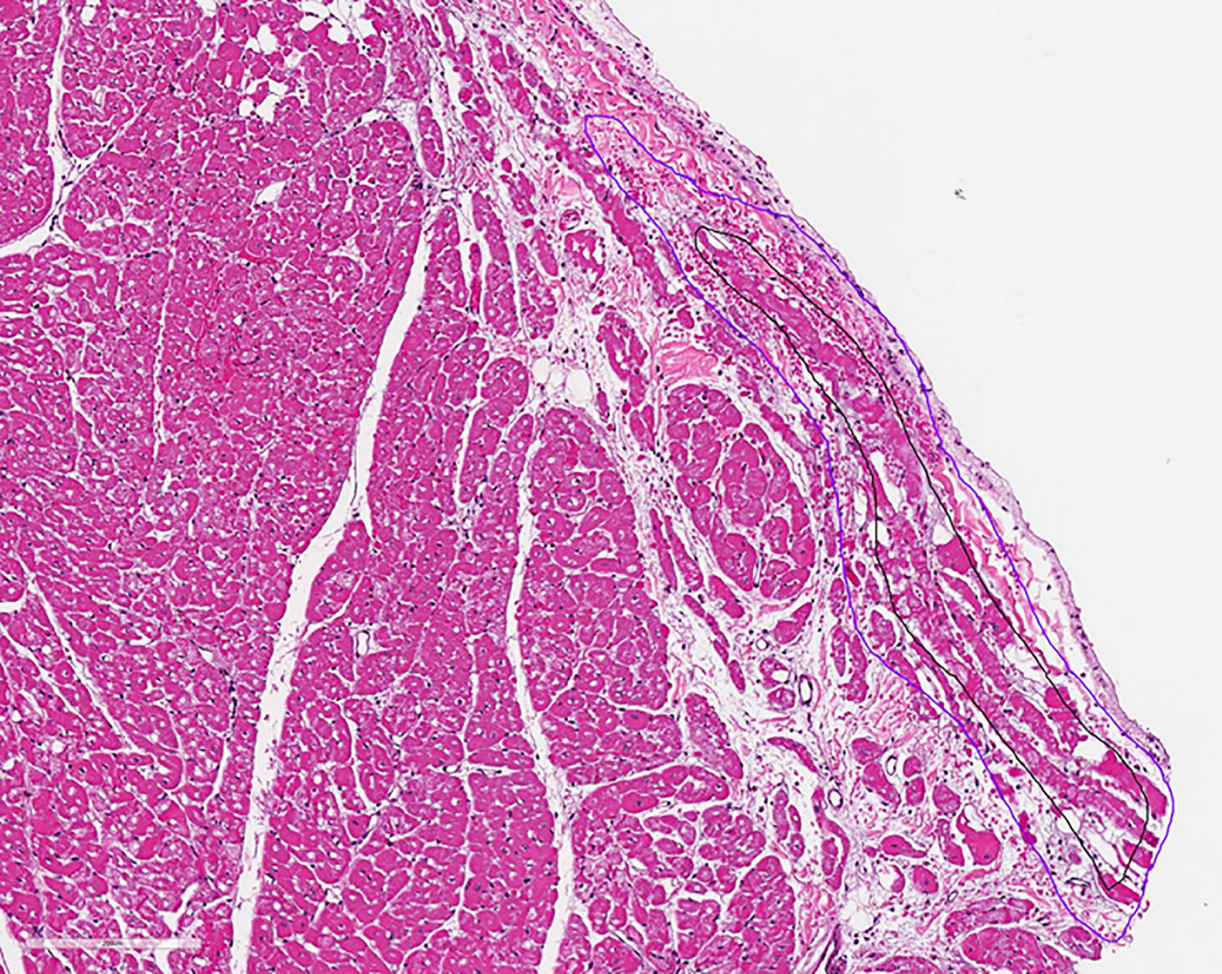
Histopathological slide showing damaged Purkinje fibers outlines in black and the purple outline demarking an area of acute hemorrhage.

In the acute studies, the histopathological analysis confirmed acute Purkinje fiber damage in 4 of the 5 studies (**Table 3, Fig 6**). Acute Purkinje fiber damage was seen as hypereosinophilic fibers that had altered structure and loss of cytoplasmic detail. We observed minimal contraction band necrosis and acute endocardial hemorrhage, suggesting minimal myocardial damage. In the one animal that did experience VT, there was mild endocardial hemorrhagic and minimal contraction necrosis. Analysis of the chronic studies showed the presence of normal Purkinje fibers, with no suggestion of Purkinje damage that had been observed in the acute studies (**Table 4**). The PEF delivered in these animals resulted in in subendocardial fibrosis with 5 of the 6 studies showing ablation sites that were through the entire subendocardial thickness. There was minimal myocardial fibrosis observed with most of the damage occurring in the endocardium.

**Table 3:** Histopathological analysis from acute animals.

Animal	Myocardial damage		Subendocardial Ablation (average percentage) *	Purkinje damage
	Contraction Necrosis	Endocardial hemorrhage		
1	None	None	No (0%)	No
2	Minimal	Mild	Yes (1.0%)	Yes
3	None	Minimal	Yes (5.0%)	Yes
4	None	Minimal	Yes (1.0%)	Yes
5	Minimal	Minimal	Yes (1.0%)	Yes

* A percentage of length of the entire endocardium in the section

**Table 4:** Histopathological analysis from the chronic animals.

Animal	Myocardial damage		Subendocardial Ablation (average percentage)*	Purkinje damage
	Endocardial fibrosis	Myocardial fibrosis		
1	Minimal	Minimal	Yes (11.7%)	No
2	None	None	No	No
3	Minimal	Minimal	Yes (15.0%)	No
4	Minimal	Minimal	Yes (24.0%)	No
5	Minimal	Minimal	Yes (17.5%)	No
6	Minimal	Minimal	Yes (11.7%)	No

* A percentage of length of the entire endocardium in the section

### Complications of PEF delivery

All animals survived and tolerated PEF delivery with only one of the 11 animals experiencing a major arrhythmic complication (acute animal 2, ventricular tachycardia). One animal (chronic animal 2) experienced an idioventricular rhythm of fascicular origin after delivery of the PEF which dissipated spontaneously and did not recur. Two other animals (acute animal 1, and chronic animal 2) had a brief (<30 sec) episode of atrial fibrillation after PEF delivery which did not recur.

## Discussion

This study highlights a novel application of PEF delivery to target cardiac Purkinje tissue, demonstrating several critical observations; 1) PEF can acutely and reversibly target Purkinje Fibers, manifesting electrically as loss of the PP and with histological analysis demonstrating acute Purkinje fiber targeting, 2) PEF Purkinje fiber targeting can be performed with minimal collateral injury to the underlying myocardium and 3) PEF can be safely delivered percutaneously to the ventricle with minimal complications.

### Selective acute targeting of cardiac tissue

In this study, we have demonstrated that in a beating heart, delivery of PEFs can provide a cardiac cell-specific approach. We observed acutely that PEF could target the Purkinje tissue without significant myocardial damage. Specifically, delivery of a PEF resulted in acute effects on cardiac conduction, manifesting electrically as loss of the PP and with histological analysis demonstrating acute Purkinje fiber targeting. Delivery of PEF did not result in any significant myocardial damage and resulted in selective endocardial surface ablation (where Purkinje fibers are located). The ability to target the Purkinje fibers in this manner represents and highlights the potential for PEF as a novel modality which could provide irreversible cell death or the enable reversible electropermeabilization, with the potential for Purkinje cell transfection. This observed approach is a welcome addition to the arena of ventricular arrhythmia management where current methods (radiofrequency or cryothermal ablation) for Purkinje arrhythmia treatment are suboptimal and not provide the potential for sparing of underlying myocardium. As we discuss below these data show that there appears to be a distinct PEF threshold difference between the Purkinje fibers and the surrounding myocardium.

The acute effects resulting in the arrest of Purkinje fiber function and the subsequent return of the PP in chronic studies we believe is indicative of the phenomenon—reversible electroporation. Reversible electroporation describes transient permeabilization of a cell whereby after application of an electric field the cell membrane reseals, and the cell subsequently restores homeostasis. It is the basis of gene transfer into cells[29, 30], delivery of drugs into cells[31–33] and electrochemotherapy[34–36]. We postulate that the return of the Purkinje signal (**Fig 4**) has the potential for significant clinical implications in novel arrhythmia management. Reversible electroporation can provide a means for delivery of a vector into the Purkinje cell/fiber or temporarily disruption of Purkinje conduction. We postulate that this opens opportunities for the development of the vaccines for arrhythmia management in the context of all Purkinje-related arrhythmias[21, 27, 37]. Additionally this also provides a means for abrupt and temporary arrest of Purkinje conduction in the acute setting of recalcitrant arrhythmias such as VF storm.

Further validation and mechanistic elucidation of this effect should entail the integration of reporter molecules that confirm cell membrane permeability[38]. An alternative hypothesis that could explain the return of the PP at 30 days is that there was the recovery of function from either regrowth and repair of the Purkinje fiber which has been reported after irreversible electroporation of sciatic nerve fibers.[39, 40] Our histological analyses did not, however, suggest new growth/Purkinje repair.

Not only is the reversible effect of PEF important but future optimization will enable the delivery of a durable irreversible ablation effect would have substantial clinical implications in arrhythmogenesis control. As discussed in the introduction, the cardiac Purkinje system plays a critical role in the genesis and maintenance of multiple ventricular arrhythmias including VF with abolition of the Purkinje fiber function, in animal models, conferring improved tolerance/resistance towards VF.[26, 28, 41] Therefore complete ablation of these fibers would present a novel method for VF ablation. Further, focal activity generated in the subendocardial Purkinje tissue has been reported to be the primary, if not the only, trigger for torsade de pointes in experimental long QT models.[41]

### Safety of PEF cardiac delivery

Use of PEFs in non-cardiac applications has resulted in both lethal and non-lethal cardiac arrhythmias [42–44]. To mitigate this risk, ECG synchronization during pulse delivery is a critical tool in ensuring that the energy is delivered during the absolute refractory period of the cardiac cycle. In this investigation, the delivery of electrical pulses was linked to a proprietary external R wave triggering device. Via R-wave detection and gating of delivery to coincide with this fiduciary marker, the device is triggered to deliver a pulse 50 milliseconds after each R wave[45, 46]. While delivery of PEF to atrial tissue and over the ventricular epicardial surface has been reported, this *in-vivo* investigation highlights that endocardial ventricular PEF delivery (at significant voltages) can be delivered safely with only one animal (acute animal 2) experiencing an arrhythmia. In this animal, we did notice a significant drop in myocardial voltage after PEF, and although histology did not show significant myocardial damage, it was more significant than what was observed in the other animals. One animal experienced an idioventricular rhythm of fascicular origin, this dissipated spontaneously and did not recur. Considering all published preclinical[8, 10, 47–49] and the recent first in human clinical data[50], it is reasonable to deduce that that direct cardiac IRE delivery within the ventricle or atrium (epicardial or endocardium) is reasonably safe (when timed with R wave).

### Tissue specificity and collateral damage

Tissue selectivity with PEF presents an entirely novel, unique, and attractive element in the management of arrhythmias. The arborizing nature of the Purkinje fibers makes them difficult to effectively target and ablate with current energy modalities (radiofrequency and cryothermal energy). Furthermore, these energy modalities cannot confer cell-specific targeting of ventricular myocardium. In this study, acute targeting of the Purkinje fibers (confirmed with loss of electrical signal and histological analysis) with minimal surrounding myocardial damage advances the applicability from largely non-cardiac, gross ablation of solid tissue into a realm of precise ventricular tissue “dissection.” The resilience of the His Bundle to the PEF may derive from the tissue selectivity of PEF, yet the penetrating segment is also encased in a collagenous/fibrous sheath[51], which we hypothesize, results in altered electrical field distribution and dispersion[52]. Although, not penetrating, and subendocardial in location, larger fascicles also retain insulation, which also will render different electroporative susceptibility to the underlying conducting tissue. This was seen, where at higher energies, successful eradication of electrical conduction occurred but returned after 5 minutes. The exact threshold for definitive, durable ablation of the His, Fascicle and Purkinje requires further elucidation. It has been reported that myocardium retains a threshold of > 375 V/cm[53], but this was defined at a pulse length of 50 microseconds and in isolated cardiac cells, not heterogeneous, beating tissue in a blood pool–all of which will likely alter field vector and delivery[54, 55]. Extrapolation is therefore not reliable for clinical applications from the in-vitro models and optimization for beating ventricular myocardium is necessary following this proof-of-concept investigation Additionally, as future studies are performed and different pulse protocols are explored, joule heating may occur in which irreversible electroporation is the goal. To achieve an irreversible effect higher voltages, greater pulses, or nanosecond pulse width may be required which could potentially result in Joule heating. Joule heating may result in loss of selectivity and therefore a tradeoff may exist between cell selective and non-selective irreversible damage. These considerations aside, this preliminary study presents inviting proof-of-concept data, which demonstrate acute differences in tissue selectivity, and whether or not increased energy delivery to permanently ablate the Purkinje will also show such selectivity remains to be determined.

### Tissue contact and the electrical field effect

Another key differentiator between currently available cardiac ablation tools and PEF catheters is the requirement for tissue contact. Compared with thermal/radiofrequency modalities, this fundamental tenet does not apply to PEF, and the field of effect allows for less precise delivery for widespread targeting of tissue. There is also a potential vulnerability for specific arrhythmia syndromes where tissue vulnerability is conferred by tissue type, and not contact, and is yet a particularly attractive feature where the tissue of interest retain distinctly different cellular membrane constituents.

### Limitations

It is essential to understand that the effect of PEF is directly related to the electric field delivered and its distribution in tissue. Although we state the overall voltage delivered, determination of the actual electric field distribution is complex and relies on tissue conductivity which was not feasible to calculate [56]. Moreover, the pulse length was not kept constant between acute and chronic studies. This may limit inference of the acute damage observed in the acute experiments into our chronic studies, however, based upon our experience over these sets of experiments; we feel acute effects on Purkinje fiber function was consistent through the chronic studies; additionally, we used a standard ablation catheter not designed of electroporation delivery; the development of an optimized bipolar device may yield different results. The Navistar catheter is a “blunt tool” for mapping fine electrical fascicular signals, and for detecting discrete changes in myocardial voltage. More subtle electrophysiological signals would be identified with the use of closer-spaced, smaller electrodes, and this is being employed in iterative work. Additionally, the use of the Navistar catheter instead of a multielectrode catheter may have resulted in missing subtle changes in voltages that said, histology confirmed that we generally had preservation of the myocardium. Finally, although we did not observe any changes in the QRS duration, we did not specifically examine for any change in Purkinje fiber conduction with analysis of the His bundle-ventricular interval pre and post-ablation. The functional significance of the PEF on the cardiac fascicular/Purkinje tissue and its relationship to arrhythmia syndromes such as ventricular fibrillation is an area that requires deeper study. Determination and validation of the models will be pivotal. These investigations were undertaken in the normal heart, and whether salutary effects persist across diseased myocardial substrates commonly seen in the patient prone to VT/VF remains to be determined.

## Conclusion

PEF can be safely delivered to the endocardium of the ventricle and at our pulsing protocol we have observed a dose-dependent cell-specific effect on ventricular myocardium. Specifically with the energy we delivered, we observed a PEF effect on electrical conduction on all left ventricular Purkinje fibers with limited damage to the underlying myocardium in canine studies. Further study and electric field modification are necessary to study reversible and irreversible effects on these fibers, as well as implications for cardiac conduction, mechanical function and especially ventricular arrhythmia initiation and propagation.

## Supporting information

S1 Table Complete pulsed electric field parameters of the delivery across all acute and chronic studies. (DOCX)

## Abbreviations

ACT: Activated Clotting Time
PEFs: Pulsed electric fields
PP: Purkinje potential
VF: Ventricular fibrillation
