# Supplementary material for: Feasibility of selective cardiac ventricular electroporation
Source: PLoS One. 2020 Feb 21;15(2):e0229214. doi: 10.1371/journal.pone.0229214 (PMC7034868; doi:10.1371/journal.pone.0229214)
Supplement: S1 Table — (DOCX) [file pone.0229214.s001.docx]

| **Supplemental Table 1- C**omplete pulsed electric field parameters of the delivery across all acute and chronic studies | | | | | |
| --- | --- | --- | --- | --- | --- |
| Animal | Delivery Number | Voltage (volts) | Pulse Duration (us) | Number of Pulses | Frequency (Hz) |
| Acute |  |  |  |  |  |
| 1 | 1 | 750 | 90 | 10 | 1 |
|  | 2 | 750 | 90 | 20 | 1 |
|  | 3 | 750 | 90 | 10 | 1 |
|  | 4 | 750 | 90 | 20 | 1 |
|  | 5 | 750 | 90 | 30 | 1 |
|  | 6 | 750 | 90 | 100 | 1 |
|  | 7 | 750 | 20 | 100 | 1 |
| 2 | 1 | 1800 | 20 | 10 | 1 |
|  | 2 | 1800 | 20 | 10 | 1 |
| 3 | 1 | 1050 | 20 | 10 | 1 |
|  | 2 | 1500 | 20 | 10 | 1 |
|  | 3 | 2250 | 20 | 10 | 1 |
|  | 4 | 3000 | 20 | 10 | 1 |
|  | 5 | 3000 | 20 | 10 | 1 |
|  | 6 | 750 | 20 | 10 | 1 |
|  | 7 | 1500 | 20 | 10 | 1 |
| 4 | 1 | 750 | 20 | 10 | 1 |
|  | 2 | 1125 | 20 | 10 | 1 |
|  | 3 | 1125 | 20 | 10 | 1 |
|  | 4 | 1500 | 20 | 10 | 1 |
|  | 5 | 1500 | 20 | 10 | 1 |
|  | 6 | 2250 | 20 | 10 | 1 |
| 5 | 1 | 2250 | 20 | 10 | 1 |
|  | 2 | 2250 | 20 | 10 | 1 |
|  | 3 | 2250 | 20 | 10 | 1 |
|  | 4 | 2250 | 20 | 10 | 1 |
| Chronic |  |  |  |  |  |
| 1 | 1 | 1125 | 90 | 10 | 1 |
|  | 2 | 1125 | 90 | 10 | 1 |
|  | 3 | 1125 | 90 | 10 | 1 |
|  | 4 | 1125 | 90 | 10 | 1 |
|  | 5 | 1125 | 90 | 10 | 1 |
|  | 6 | 1125 | 90 | 10 | 1 |
| 2 | 1 | 500 | 90 | 10 | 1 |
|  | 2 | 1050 | 90 | 10 | 1 |
|  | 3 | 750 | 90 | 10 | 1 |
|  | 4 | 750 | 90 | 10 | 1 |
|  | 5 | 750 | 90 | 10 | 1 |
|  | 6 | 1500 | 90 | 10 | 1 |
|  | 7 | 1500 | 90 | 10 | 1 |
| 3 | 1 | 750 | 90 | 10 | 1 |
|  | 2 | 750 | 90 | 10 | 1 |
|  | 3 | 1050 | 90 | 10 | 1 |
|  | 4 | 1050 | 90 | 10 | 1 |
|  | 5 | 1050 | 90 | 10 | 1 |
| 4 | 1 | 750 | 90 | 10 | 1 |
|  | 2 | 750 | 90 | 10 | 1 |
|  | 3 | 1125 | 90 | 10 | 1 |
|  | 4 | 1125 | 90 | 10 | 1 |
| 5 | 1 | 750 | 90 | 10 | 1 |
|  | 2 | 750 | 90 | 10 | 1 |
| 6 | 1 | 1500 | 90 | 10 | 1 |
